# LONGITUDINAL STUDY OF R1RHO DISPERSION IMAGING OF AGING MUSCLE IN FISCHER RATS

**DOI:** 10.1093/geroni/igae098.2295

**Published:** 2024-12-31

**Authors:** Fatemeh Adelnia, Feng Wang, John Gore

**Affiliations:** Vanderbilt University Medical Center, Nashville, Tennessee, United States; Vanderbilt University Medical Center, Nashville, Tennessee, United States; Vanderbilt University, Nashville, Tennessee, United States

## Abstract

Aging is associated with impaired endothelium-dependent vasodilation, leading to impaired muscle perfusion and organ dysfunction. It has been shown that impaired muscle perfusion may result in insufficient delivery of oxygen and nutrients during and after muscle contraction, leading to muscle damage. The ability to study the relationships between microvascular structure, impairment of perfusion, and muscle damage has been limited using traditional muscle perfusion measures, which are mostly invasive and risky. We developed a non-invasive 3D R1ρ dispersion MRI method at weak locking field frequencies to quantify microvasculature restructuring in skeletal muscle. This method provides a unique way of characterizing the size and density of capillaries. We successfully developed the protocol and applied it to the lower leg of F334 female rats of different ages. The whole lower leg of each rat was also imaged using conventional RARE pulse sequences to quantify the T2 values of the muscles. Our data show ΔR1ρ (=R1ρ{0Hz} - R1ρ{240Hz}) fraction values in calf muscles of 15 months F344 female rats (n=8) are significantly (p-value=0.03) higher than 6 months old rats (n=8). We interpret this measure as a possible new biomarker that characterizes changes in microvascular structure with aging. The T2 value also significantly increased in older rats (=30.25ms) compared to younger groups (=27.7ms), possibly reflecting higher inflammation in muscle in agreement with previously published results. The future direction of this ongoing study is to compare these results with data collected from male rats and validation of the ΔR1ρ fraction as a biomarker using ex-vivo histology.

